# The future of the development of medicines in idiopathic pulmonary fibrosis

**DOI:** 10.1186/s12916-015-0480-7

**Published:** 2015-09-24

**Authors:** Laura Fregonese, Irmgard Eichler

**Affiliations:** Orphan Medicines Office, European Medicines Agency, 30 Churchill Place, Canary Wharf, E14 5EU London, UK; Paediatric Medicines Office, European Medicines Agency, London, UK

**Keywords:** Clinical development, Drug discovery, Early access, Idiopathic pulmonary fibrosis, Outcome measures, Regulatory approval

## Abstract

The development of treatments for idiopathic pulmonary fibrosis (IPF) has been often disappointing. Building on authorized treatments that can benchmark the validity of treatment effect measures, the time has come to standardize endpoints and achieve consensus on their use for different clinical questions and specific IPF phenotypes. In order to facilitate the development of new medicines for IPF it is crucial that the knowledge of the disease and lessons learnt from past trials are taken forward to create international trial networks with involvement of patients, including biobanks and clinical data collection through a multinational registry. Interaction with regulators may be useful to align the initiatives of academia and pharmaceutical companies with the bodies ultimately responsible for licensing new products. Interaction can occur through the use of qualification programs for biomarkers and endpoints, and participation in innovative regulatory pathways and initiatives. Finally, the experience of IPF should be used to benefit even rarer interstitial lung diseases for which no treatment is available, including pediatric interstitial lung diseases. This commentary provides a perspective on the hurdles slowing the development and regulatory approval of medicines for IPF, and encourages close cooperation between investigators and drug regulators.

## Background

The development of new medicines is characterized by very high attrition rates, with up to 10,000 compounds failing to show clinical efficacy per each new medicine reaching the market. Failure occurs mainly during translation from preclinical to clinical development and in clinical phase II, with efficacy rather than safety the most common cause of attrition [1, 2]. The respiratory field is no exception, with one of the lowest numbers of new medicines authorized in the past 40 years among all medical fields [3].

In this landscape, until recently no pharmacological treatment was available for idiopathic pulmonary fibrosis (IPF), a rare disease of largely unknown cause with median survival estimated at about 3 years from diagnosis [4]. After a decade of failed clinical development, pirfenidone (Esbriet) obtained marketing authorization in Europe in February 2011 and nintedanib (OFEV) in 2015. Past failures have been attributed to the heterogeneous pathogenesis of the disease, the inappropriateness of study designs and endpoints used in clinical trials [5–7], and the lack of merit of the products studied, which in many cases were repurposed and not specifically developed for IPF.

While pirfenidone and nintedanib have proven to slow the decline of lung function in the clinical trials on which their approval was based [8], their effects on the long-term prognosis of the disease are still unknown. This, together with the emerging evidence of different phenotypes in IPF [9], and the lack of treatments for interstitial lung diseases (ILDs) other than IPF, renders it an area of highly unmet therapeutic need.

## IPF drug discovery between present and future

To date, 12 medicinal products, most in preclinical phase of development, have obtained orphan designation from the European Medicines Agency (EMA) [10] for the treatment of IPF. While not compulsory, it is in the interest of companies to obtain orphan designation, because it allows them to apply for clinical development incentives and for fee reductions in regulatory procedures. The number of designated medicines, although not a proxy of pharmaceutical development in a strict sense, provides a trend of the development in specific diseases. The number of orphan designations for IPF compares unfavorably with that of other rare respiratory diseases such as cystic fibrosis, with 42 designations [11], and pulmonary arterial hypertension, where six out of 15 products designated as orphans in the EU in the past ten years have already obtained marketing authorization [12].

Traditionally, pharmaceutical development starts with the selection of scientifically robust therapeutic targets and of the compounds with the highest activity on the selected target(s). While this may still be valid in diseases characterized by good correlation between genotype, molecular phenotype, and clinical phenotype, in complex trait diseases such as IPF, determined by multiple genes and variable influence by the environment, drug discovery tools commonly used to select and validate targets at preclinical level, such as high throughput compound profiling in cell-based assays [13, 14], may prove unsuccessful.

Alternative approaches for identifying therapeutic targets in IPF should be encouraged, including those based on system biology and integrative omics. So far a handful of studies have investigated omics evidence in IPF, with hypothesis-generating results such as the discovery of genetic variants associated with alterations of lung host defense [15] (microbiome), of transcriptional effects of autoantibodies against collagen V [16] (“antibodome”), and of genomic and histology-based specific patterns of disease [17]. Innovative drug development would require data knowledge integration, connecting genetic and molecular aspects of IPF with the network of downstream events and the clinical manifestations of the disease and its progression. It is worth broadly applying integrative approaches to progressive pulmonary fibrosis [18] rather than limiting them to IPF, because many pathogenetic aspects are common to different disease entities characterized by pulmonary fibrosis, including the role of epithelial cells, DNA repair and cell senescence, and the endoplasmic reticulum [15, 19].

With translational failure of many IPF pharmaceutical pipelines, recurrent criticism arises toward the most widely used in vivo preclinical proof of concept, the bleomycin challenge rodent model. Indeed, none out of almost 300 compounds demonstrating efficacy in the bleomycin model was then efficacious in clinical trials [20]. The model was, however, used in the preclinical development of pirfenidone [21] and nintedanib [22], compounds with a successful clinical development. To clarify the usefulness and limitations of the bleomycin model as proof of concept in IPF and other ILDs, standardization is needed of methodological aspects, such as the timing of administration of candidate products—which are currently administered in most preclinical studies before or immediately after bleomycin challenge [20] as opposed to the clinical situation where lung fibrosis is already present at the start of treatment. At the same time, there is urgent need of preclinical proof-of-concept tools going beyond animal models to include the use of human tissue-based and system biology readouts.

## The future of clinical development in IPF and beyond

In addition to the complexity induced by pathogenetic and clinical heterogeneity, the development of medicines for the treatment of IPF suffers from the hurdles characteristic of rare diseases, where small numbers of patients limit the feasibility of adequately powered trials, the validation of endpoints and biomarkers [23], and the use of hard endpoints such as mortality. From a regulatory perspective, additional factors complicating the clinical development in IPF include changes over time in case definition and lack of consistency in clinical trial endpoints.

The recent consensus on imaging-based diagnosis [24] may facilitate the conduct of IPF trials in clearly defined study populations. On the other hand, the great heterogeneity in outcome measures used across clinical trials has largely limited the feasibility to conclusively assess the validity of most of them. Indeed, while slowing the decline of forced vital capacity was accepted as the primary endpoint for the regulatory approval of pirfenidone and nintedanib [8], there is not yet consensus on its minimal clinically important difference [25]. Mortality, used mostly as a secondary endpoint, has also been measured inconsistently in IPF trials, as all-causes mortality, IPF-specific mortality, time to death, progression-free survival, or survival time [7]. Finally, composite endpoints have been suggested as a way to improve trial efficiency and sensitivity to drug effect [7]; however, potentially interesting measures in composite endpoints, such as symptoms and clinical worsening, have not been validated in IPF.

A recent consensus group proposing provisional core sets of domains and instruments for clinical trials in connective tissue disease-related ILDs and IPF concluded that none of the proposed endpoints were ideal, or fully validated [26]. Global collaborative work for validation and standardization of endpoints for regulatory use is needed to facilitate the clinical development and the approval of new medicines in IPF. This is warranted by the increasing complexity of clinical trials, where efficacy will now have to be established on top of, or in comparison to, the authorized treatments [27]. The consistent use of standardized endpoints across trials would improve comparability of compounds during pre-licensing clinical development, and provide continuity in the measurement of treatment effects through the whole lifecycle of medicinal products. This would allow the harmonization of the assessment of regulatory, health technology assessment, and payers’ bodies on the therapeutic added value of different products, and increase clarity on the clinical benefit for prescribing physicians and patients.

Ideally, outcome measures would be chosen from among those that can be monitored in patients’ registries, to align measures of natural history of the disease with disease progression parameters measurable in clinical trials. Initiatives are ongoing at EMA to explore the use of registry data in the frame of regulatory procedures [28], in particular for the establishment of real-life post-approval confirmation of effectiveness. A handful of national IPF registries exist in Europe [29]; however, a European rather than national dimension would be needed, ideally integrated with the recently proposed initiatives of an IPF clinical trial network [30]. The participation of patients in international disease networks is crucial to the development of patient-reported outcome measures, at present poorly developed in IPF, and in line with current initiatives incorporating patients’ views into the benefit–risk assessment of medicines.

Among the initiatives facilitating the approval of new medicines, early access regulatory pathways, such as the adaptive pathway in pilot phase in Europe [31], aim to optimize development by balancing the need for timely patient access with the importance of providing adequate, evolving information on a medicine’s benefit and risk, including collection of real-world data post-authorization to complement randomized clinical trials data. IPF appears to be a potential candidate in this respect, because the development of medicines initially developed for IPF could be gradually expanded to other target populations, e.g., ILDs other than IPF, for most of which currently no treatment exists, including pediatric ILDs.

## Conclusions

Global collaborative efforts are warranted to facilitate the development of new medicines for IPF, including data knowledge integration from genomics to clinical phenotypes. Lessons from past clinical trials should be used to create international trial networks with involvement of patients, including data collection through biobanks and a European registry, and for standardization of outcome measures. Interaction with the regulators for fostering the approval of new medicines can occur through qualification programs for biomarkers and endpoints, and participation in innovative regulatory pathways and initiatives. Finally, the experience of IPF should be applied to the development of medicines for ILDs for which no treatment is available, including pediatric ILDs.

## Abbreviations

EMA: European Medicines Agency
ILDs: interstitial lung diseases
IPF: idiopathic pulmonary fibrosis
